# Supplementary material for: Remodeling Membrane Binding by Mono-Ubiquitylation
Source: Biomolecules. 2019 Jul 31;9(8):325. doi: 10.3390/biom9080325 (PMC6723200; doi:10.3390/biom9080325)
Supplement: Supplementary file 1 [file biomolecules-09-00325-s001.pdf]

MSKQFVR, MH+ 1009.5253, m/z 505.2663
UbEnt1.02092.02092.2.dta

K3

b+	#	AA	#	y+	y2+
132.0483	1	M	7		
219.0803	2	S	6	878.4848	439.7463
461.2182	3	K	5	791.4528	396.2303
589.2768	4	Q	4	549.3149	275.1614
736.3452	5	F	3	421.2563	211.1321
835.4136	6	V	2	274.1879	137.5979
	7	R	1	175.1195	88.0637

K(3):+242.14

scan 2092; 1.6e+03

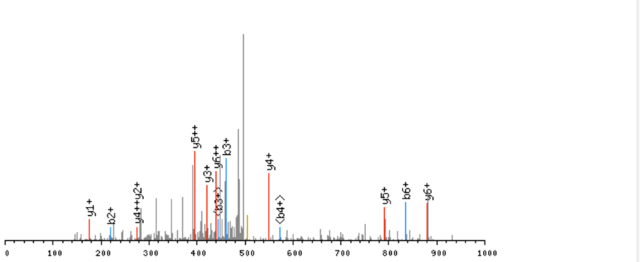

NLVKGYSSTQVLVR, MH+ 1677.9288, m/z 559.9811
Seq26286_E101.03623.03623.3.dta

K14

b+	b2+	#	AA	#	y+	y2+	y3+
115.0508	58.0293	1	N	14			
228.1348	114.5713	2	L	13	1563.8859	782.4469	521.9672
327.2032	164.1055	3	V	12	1450.8018	725.9048	484.2725
569.3411	285.1745	4	K	11	1351.7334	676.3706	451.2497
626.3626	313.6852	5	G	10	1109.5955	555.3017	370.5370
789.4259	395.2169	6	Y	9	1052.5740	526.7909	351.5299
876.4580	438.7329	7	S	8	889.5107	445.2593	297.1755
963.4900	482.2489	8	S	7	802.4787	401.7433	268.1648
1064.5377	532.7727	9	T	6	715.4466	358.2272	239.1541
1192.5962	596.8020	10	Q	5	614.3990	307.7034	205.4715
1291.6646	646.3362	11	V	4	486.3404	243.6741	162.7853
1404.7487	702.8783	12	L	3	387.2720	194.1399	129.7625
1503.8171	752.4125	13	V	2	274.1879	137.5979	92.0679
		14	R	1	175.1195	88.0637	59.0451

K(4):+242.14

scan 3623; 5.0e+02

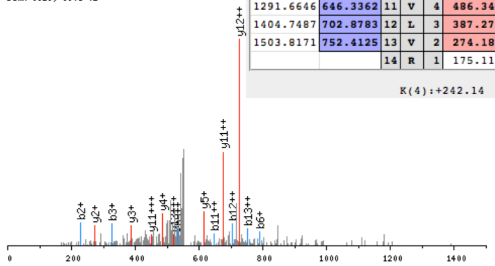

ENLYIIKTLK, MH+ 1348.7840, m/z 674.8957
Seq26286_E101.04287.04287.2.dta

K103

b+	#	AA	#	y+	y2+
130.0504	1	E	10		
244.0933	2	N	9	1219.7414	610.3746
357.1774	3	L	8	1105.6985	553.3532
520.2407	4	Y	7	992.6145	496.8111
633.3248	5	I	6	829.5511	415.2795
746.4089	6	I	5	716.4671	358.7374
988.5468	7	K	4	603.3830	302.1954
1089.5944	8	T	3	361.2451	181.1265
1202.6785	9	L	2	260.1974	130.6026
	10	K	1	147.1134	74.0606

K(7):+242.14

scan 4287; 5.1e+02

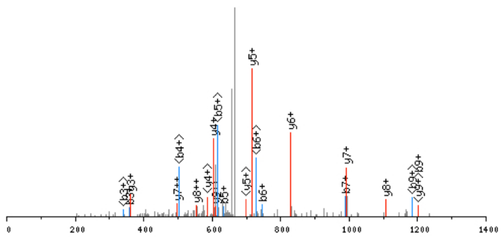

Supplementary Figure 1. Mass-spectrometry analyses of purified ubiquitylated-Ent1 derivatives from *E. coli* based expression system.

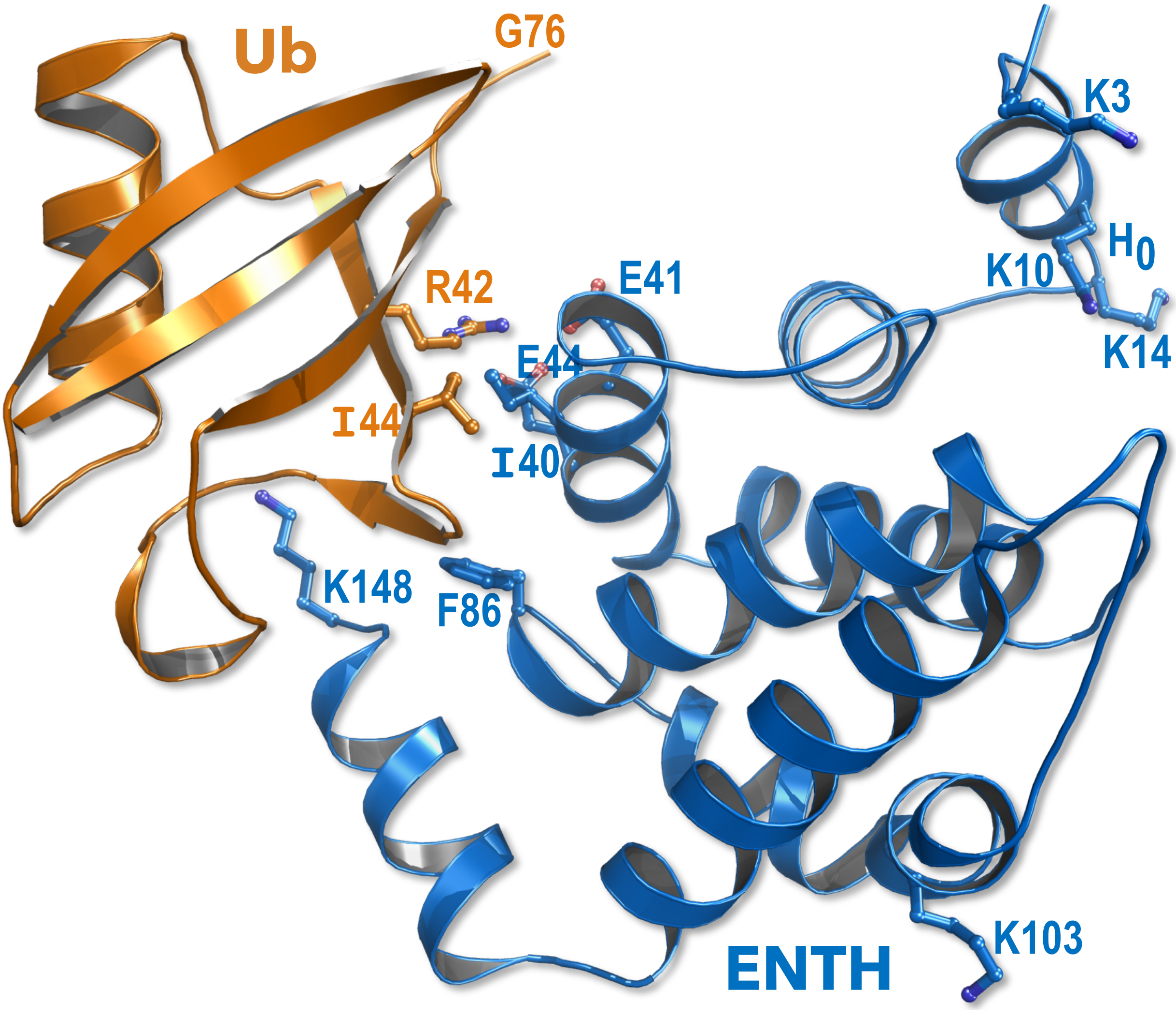

Supplementary Figure 2. Structural model of ENTH:Ub complex. We superimposed the yeast ENTH on the STAM-VHS:Ub complex (3LDZ) and then use Fiberdoc for flexible induced-fit backbone refinement [39]. The residues at the binding interface and the lysine residues that undergo ubiquitylation are shown as sticks.

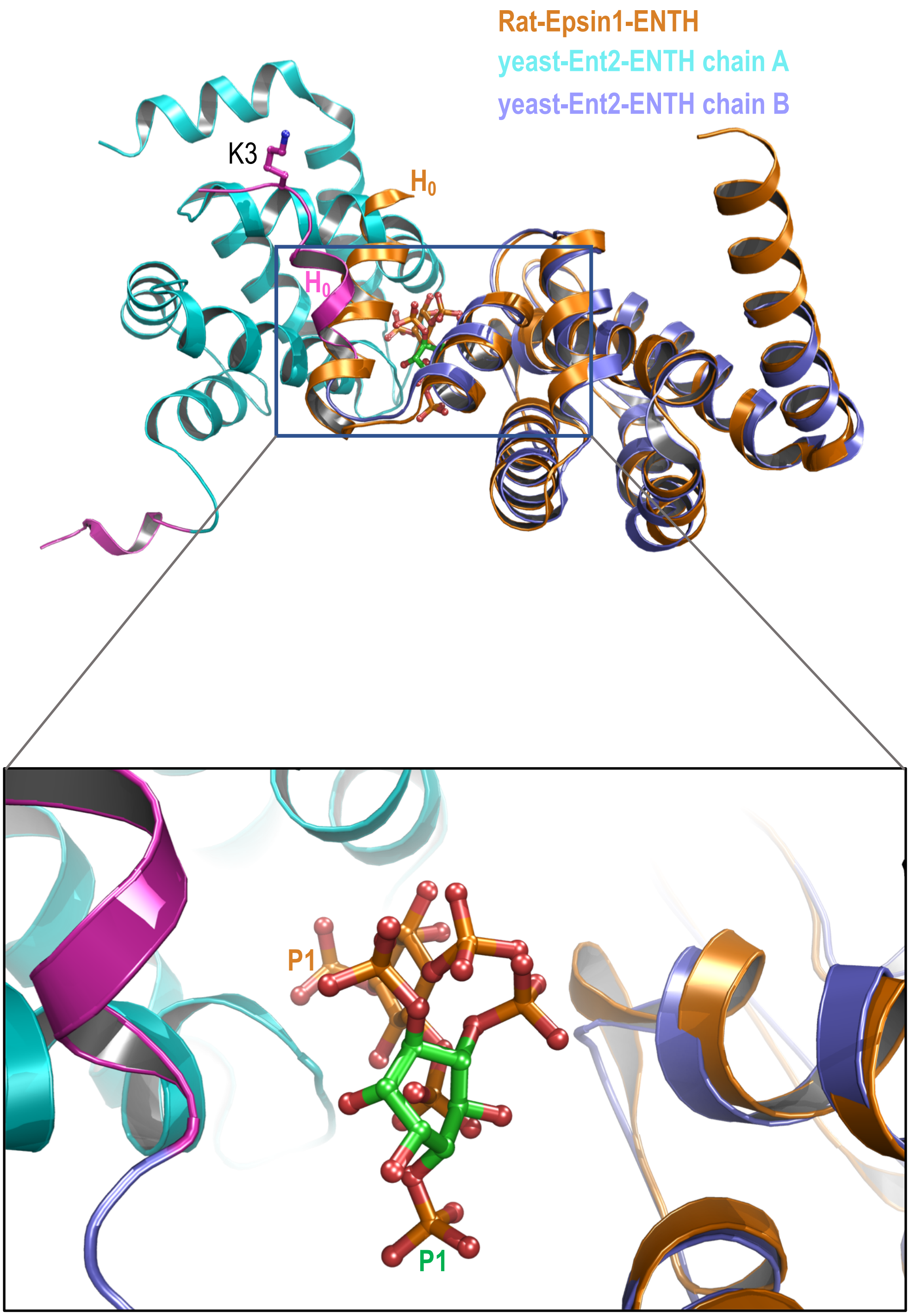

Supplementary Figure 3. structure of yeast Ent2 homo dimer super imposed with rat Epsin1 ENHT domains. Top shows the overall structure of the proteins domain. The changes in the orientation of H_0 is shown. Bottom, is a zoom-in into the ligand binding site, shows the different location and orientation of the PIP_2 molecules. The orange PIP_2 is from the rat-ENTH complex and the green molecule is from the Ent2 complex.
